# Pancreaticoduodenectomy in patients < 75 years versus ≥ 75 years old: a comparative study

**DOI:** 10.1007/s40520-024-02804-9

**Published:** 2024-07-04

**Authors:** Muhammer Ergenç, Tevfik Kıvılcım Uprak, Ayşegül Bahar Özocak, Şakir Karpuz, Mümin Coşkun, Cumhur Yeğen, Ali Emre Atıcı

**Affiliations:** 1 Department of General Surgery, Marmara University School of Medicine, Başıbüyük Campus Başıbüyük Mah. Maltepe Başıbüyük Yolu Sok. No: 9/2 Maltepe 34854, Istanbul, Turkey; 2 Department of General Surgery, Hınıs Şehit Yavuz Yürekseven State Hospital, Yenikent Mahallesi Cumhuriyet Caddesi No:7/4 Hınıs, 25600 Erzurum, Turkey

**Keywords:** Elderly, Geriatric surgery, Pancreatic adenocarcinoma, Pancreatectomy, Performance status, Survival

## Abstract

**Objective:**

This study aimed to compare the postoperative outcomes of < 75-year-old patients and ≥ 75-year-old patients who underwent pancreaticoduodenectomy (PD) for pancreatic head and periampullary region tumors.

**Methods:**

Patients who underwent PD in our hospital between February 2019 and December 2023 were evaluated. Demographics, Eastern Cooperative Oncology Group Performance Status (ECOG-PS) scores, American Society of Anesthesiologists (ASA) scores, comorbidities, hospital stays, complications, and clinicopathological features were analyzed. Patients were divided into < 75 years (Group A) and ≥ 75 years (Group B) groups and compared.

**Results:**

The median age of the entire cohort (n = 155) was 66 years (IQR = 16). There was a significant difference between Group A (n = 128) and Group B (n = 27) regarding the ECOG-PS and ASA scores. There was no significant difference between the groups regarding postoperative complications. The 30-day mortality rate was greater in Group B (p = 0.017). Group B had a cumulative median survival of 10 months, whereas Group A had a median survival of 28 months, with a statistically significant difference (p < 0.001). When age groups were stratified according to ECOG-PS, for ECOG-PS 2–3 Group A, survival was 15 months; for ECOG-PS 2–3 Group B, survival was eight months, and the difference was not statistically significant (p = 0.628).

**Conclusions:**

With the increasing aging population, patient selection for PD should not be based solely on age. This study demonstrated that PD is safe for patients older than 75 years. In older patients, performance status and the optimization of comorbidities should be considered when deciding on a candidate’s suitability for surgery.

## Introduction

Pancreatic cancer is a cancer with a poor prognosis that causes a significant number of deaths worldwide. It usually has an insidious onset and is detected at an advanced stage. The incidence and mortality increase with increasing age. Pancreatic adenocarcinoma accounts for 85% of pancreatic cancers, and the majority are diagnosed in patients over the age of 70. The incidence and mortality of pancreatic cancer have increased over the past thirty years in numerous nations, affecting both sexes and various age groups [1–3].

The main treatment for pancreatic cancer is margin-negative (R0) surgical resection. Chemotherapy, radiotherapy, and their combinations are used. Age is not a contraindication for surgical resection and has been extensively studied. However, the optimal treatment regimen for older persons remains controversial. The challenge in treating older persons is accurately identifying patients who can tolerate more aggressive treatment regimens with less morbidity [3–8].

Improvements in health conditions in recent decades have resulted in longer human lifespans. However, aging has also led to an increase in the rate of age-related chronic diseases and disability. Inevitably, with this demographic change, the number of conditions requiring surgery in the older population is increasing. This has gradually changed the definition of an older person for surgeons, with an increasing number of complex surgeries being performed even for patients over the age of 85. Assessing whether a patient is too old for surgery can be challenging, and advancing age alone can significantly increase the chances of complications and mortality during and after the operation. Based only on the raw statistics, there is a clear and substantial rise in mortality as age increases. However, when considering additional factors, including physiological changes, coexisting medical conditions, types of surgery, and the timing of surgical intervention, the findings vary. Studies have clearly shown that age itself is not a prognostic risk factor for complications after elective surgery in older patients, while cognitive or functional frailty is. Both the referring physician and the assessing surgeon should not refuse surgery to patients based merely on their age. Instead, judgments should be made based on a comprehensive geriatric assessment that considers the patient’s cognitive, functional, nutritional, socioeconomic, and emotional condition [9–15].

As the demographics of many countries are moving toward an aging population, older pancreatic cancer patients may be denied surgical treatment solely due to the perceived risks associated with chronological age. Multiple studies have presented conflicting findings on surgical results following pancreaticoduodenectomy (PD) or pancreatic resection in older patients. Studies often show increased morbidity rates in older individuals compared to younger people. When examining mortality, certain studies indicate no disparity, but others suggest increased mortality in the older age range. Not only chronological age but also patient frailty and performance status influenced these results. However, there are differences between the populations defined as elderly in the studies. Therefore, the indications and outcomes of pancreatic surgery for patients ≥ 75 years old need to be investigated [16–21].

The purpose of this research was to compare the postoperative outcomes of < 75-year-old patients and ≥ 75-year-old patients who underwent pancreaticoduodenectomy for tumors of the pancreatic head and periampullary region.

## Materials and methods

The data of patients who underwent pancreatic surgery at Marmara University Hospital and were subsequently followed up in the general surgery clinic between February 2019 and December 2023 were analyzed.

The investigation was granted approval by the Clinical Research Ethics Committee of the Marmara University School of Medicine (Number: 09.2024.74).

Patients who underwent pancreatic surgery other than PD surgery between these dates were excluded. After excluding patients whose operative and postoperative data could not be accessed and whose follow-up data were missing, the remaining patients were included in the study.

Demographics, body mass index, Eastern Cooperative Oncology Group Performance Status (ECOG-PS) scores, American Society of Anesthesiologists (ASA) scores, preoperative biliary drainage status, bilirubin levels, tumor markers, preoperative symptoms, comorbidities, neoadjuvant treatment status, operative time, amount of bleeding, intensive care unit stay, hospital stay, postoperative hospital stay, complications, and clinicopathological features of the tumors were analyzed.

Patients who underwent conventional PD after resectability were evaluated by excluding hepatic, peritoneal, or other distant metastases. The Blumgart or Heidelberg technique was used for pancreatic anastomosis.

Patient performance status was assessed using the ECOG PS, a synthesized scale of symptoms and mobility that has been in clinical use for a long time [22].

The tumor stages were categorized based on the American Joint Committee on Cancer (AJCC) guidelines [23]. The Clavien–Dindo classification was used to assess postoperative complications [24]. Pancreatic fistula, delayed gastric emptying (DGE), and hemorrhage following pancreatectomy were classified based on the International Study Group of Pancreatic Surgery criteria [25–27].

Based on previous studies and the World Health Organization classification, individuals aged 75 years and older were defined as elderly [28–31].

Patients were categorized into two groups based on age: less than 75 years (Group A) and 75 years and older (Group B). All parameters were then compared between the two groups.

### Statistical analysis

The statistical analyses were conducted using the Statistical Package for the Social Sciences (SPSS, Version 24 for Mac), developed by IBM Corporation. Normally distributed data are presented as the mean ± standard deviation, while nonnormally distributed data are presented as median (interquartile range, IQR) values. The chi-square test was utilized to compare categorical data. Student’s t test was used to compare parametric data, whereas the Mann‒Whitney U test was used to compare nonparametric data. The Kaplan‒Meier method was utilized for survival analysis, and the log-rank test was used for univariate analysis. A 95% confidence interval and a two-sided p value < 0.05 were considered to indicate statistical significance.

## Results

During the study, 228 patients underwent pancreatic resection for various malignant and benign diseases in our clinic. We excluded 44 patients who underwent distal pancreatectomy, four patients who underwent radical antegrade modular pancreatosplenectomy, five patients who underwent chronic pancreatitis procedures, 15 patients who underwent total pancreatectomy, and five patients whose follow-up data were unavailable. Data from 155 patients who underwent PD were analyzed. Patients were divided into 128 patients (Group A) aged < 75 years and 27 patients (Group B) aged ≥ 75 years for comparison.

The median age of the entire cohort was 66 years (IQR = 16). The ages of the patients ranged between 25 and 85 years. Age, sex, preoperative data, and comorbidities are shown in Table 1. There was a significant difference between groups regarding the ECOG-PS score, ASA score, incidence of chronic kidney disease, and incidence of hypertension. However, no significant differences in the other parameters were observed between the two groups.

**Table 1:** Demographic and clinical features

Variables	All patients	Group A < 75 years	Group B ≥ 75 years	p value
	N = 155	n = 128	n = 27	
Age (years) (median, IQR)	66 (16)	63 (49)	78 (5)	** < 0.001**
Sex (n, %)				
Female	67 (43.2)	52 (40.6)	15 (55.6)	0.155
Male	88 (56.8)	76 (59.4)	12 (44.4)	
BMI (kg/m^2^) (mean ± SD)	26.6 ± 5.6	26.8 ± 4.5	26.7 ± 3.9	0.608
ECOG-PS (n, %)				
0–1	135 (87.1)	118 (92.2)	17 (63)	** < 0.001**
2–3	20 (12.9)	10 (7.8)	10 (37)	
ASA Score (n, %)				
1	15 (9.7)	14 (10.9)	1 (3.7)	** < 0.001**
2	69 (44.5)	65 (50.8)	4 (14.8)	
3	71 (45.8)	49 (38.3)	22 (81.5)	
Preoperative biliary drainage (n, %)				
ERCP	71 (45.8)	58 (45.3)	13 (48.1)	0.497
PTBD	35 (22.6)	28 (21.9))	7 (25.9)	
ERCP + PTBD	2 (1.3)	1 (0.8)	1 (3.7)	
Total bilirubin (median, IQR)	1.79 (4.8)	1.64 (4.9)	2.55 (4.5)	0.901
Direct bilirubin (median, IQR)	1 (3.1)	0.68 (3.2)	1.5 (2.9)	0.969
CEA (ng/mL) (median, IQR)	2.1 (3.4)	2.13 (4.5)	2.1 (3.5)	0.297
CA19-9 (U/mL) (median, IQR)	34.7 (230)	35.7 (176)	26 (338)	0.288
Symptoms (n, %)				
Jaundice	103 (66.5)	82 (64.1)	21 (77.8)	0.170
Weight loss	52 (33.5)	46 (35.9)	6 (22.2)	0.170
Epigastric pain	77 (49.7)	63 (49.2)	14 (51.9)	0.804
Pancreatitis	5 (3.2)	5 (3.9)	0	0.588
Comorbidities (n, %)				
Congestive heart failure	12 (7.7)	12 (9.4)	0	0.091
Coronary artery disease	26 (16.8)	23 (18)	3 (11.1)	0.290
Hypertension	69 (44.5)	48 (37.5)	21 (77.8)	** < 0.001**
Diabetes mellitus	59 (38.1)	45 (35.2)	14 (51.9)	0.104
COPD	11 (7.1)	8 (6.3)	3 (11.1)	0.295
Chronic Kidney Disease	4 (2.6)	1 (0.8)	3 (11.1)	**0.017**
Others	65 (41.9)	52 (40.6)	13 (48.1)	0.472
Neoadjuvant chemotherapy (n, %)	4 (2.6)	4 (3.1)	0	0.461

A p-value < 0.05 was considered significant

*IQR* interquartile range, *SD* standard deviation, *ASA* American Society of Anesthesiologists physical status, *BMI* body mass index, *CA19-9* carbohydrate antigen 19–9, *CEA* cancer embryonic antigen, *ECOG-PS* eastern cooperative oncology group performance status scale, *COPD* chronic obstructive pulmonary disease, *ERCP* endoscopic retrograde cholangiopancreatography, *PTBD* percutaneous transhepatic biliary drainage

As shown in Table 2, there was no difference between the two groups in terms of intraoperative data. The total length of hospital stay was significantly greater in Group B (p = 0.007), while there was no difference in postoperative hospital stay (p = 0.784). There was no significant difference between the two age groups in terms of postoperative complications. The 30-day mortality rate was greater in Group B (p = 0.017).

**Table 2:** Comparison of postoperative complications and clinical outcomes

Variables	All pateints	Group A < 75 years	Group B ≥ 75 years	p value
	N = 155	n = 128	n = 27	
Operation time (min) (median, IQR)	215 (40)	210 (40)	240 (62)	0.907
Combined resection of other organs (n, %)	6 (3.9)	4 (3.1)	2 (7.4)	0.280
Combined resection of portal vein (n, %)	0	0	0	
Intraoperative blood loss (mL) (median, IQR)	500 (348)	500 (400)	500 (200)	0.677
Intraoperative blood transfusion (n, %)	25 (16.1)	20 (15.6)	5 (18.5)	0.774
Intensive care unit admission (n, %)	23 (14.8)	16 (12.5)	7 (25.9)	0.131
Length of hospital stay (days) (median, IQR)	9 (3)	8 (3)	10 (5)	**0.007**
Length of postoperative hospital stay (days) (median, IQR)	8 (3)	8 (3)	8 (3)	0.784
Morbidity (Clavien‒Dindo ≥ 3a) (n, %)	40 (25.8)	33 (25.8)	7 (25.9)	0.988
Complications (n, %)				
Pancreatic Fistula				
Grade A	37 (23.9)	30 (23.4)	7 (25.9)	0.943
Grade B	16 (10.3)	13 (10.2)	3 (11.1)	
Reoperation	4 (2.6)	4 (3.1)	0	0.461
Delayed gastric emptying	43 (27.7)	35 (27.3)	8 (29.6)	0.809
Bile leakage	5 (3.2)	4 (3.1)	1 (3.7)	0.621
Intraabdominal abscess	12 (7.7)	9 (7)	3 (11.1)	0.440
Bleeding	7 (4.5)	7 (5.5)	0	0.606
Postoperative Blood Transfusion	19 (12.3)	18 (14.1)	1 (3.7)	0.200
30-day mortality (n, %)	4 (2.6)	1 (0.8)	3 (11.1)	**0.017**

A p-value < 0.05 was considered significant

The detailed postoperative pathologic data are shown in Table 3. When the pathology results were grouped as malignant or benign, no difference was observed between the two groups. There was no significant difference between the two groups in terms of the node-positive disease rate or T stage.

**Table 3:** Comparison of pathologic features

Variables	All patients	Group A < 75 years	Group B ≥ 75 years	p value
	N = 155	N = 128	N = 27	
Pathology (n, %)				
Malignant	140 (90.3)	115 (89.8)	25 (92.6)	0.494
Benign	15 (9.7)	13 (10.2)	2 (7.4)	
Malignant pathology (n, %)				
Ductal adenocarcinoma	115 (74.2)	95 (74.2)	20 (74.1)	
Intraductal papillary mucinous neoplasm with an associated invasive carcinoma	2 (1.3)	2 (1.6)	0	
Neuroendocrine tumor	6 (3.9)	6 (4.7)	0	
Mucinous cystic neoplasms with an associated invasive carcinoma	3 (1.9)	3 (2.3)	0	
Solid pseudopapillary neoplasm	1 (0.6)	1 (0.8)	0	
Duodenal adenocarcinoma	1 (0.6)	0	1 (3.7)	
Adenosquamous carcinoma	2 (1.3)	2 (1.6)	0	
Acinar cell carcinoma	1 (0.6)	0	1 (3.7)	
Anaplastic undifferentiated carcinoma	1 (0.6)	1 (0.8)	0	
Other malignant	9 (5.8)	6 (4.7)	3 (11.1)	
Benign pathology (n, %)				
Chronic pancreatitis	3 (1.9)	3 (2.3)	0	
Autoimmune pancreatitis	3 (1.9)	3 (2.3)	0	
Cystadenomas	2 (1.3)	2 (1.6)	0	
Groove pancreatitis	2 (1.3)	2 (1.6)	0	
Other benign	3 (1.9)	1 (0.8)	2 (7.4)	
Tumor size (mm) (median, IQR)	30 (19)	30 (18)	35 (22)	0.577
Number of examined lymph nodes (median, IQR)	21 (12)	23 (12)	20 (11)	0.138
Positive lymph nodes (median, IQR)	3 (06)	3 (6)	3 (4)	0.425
Lymphovascular invasion (n, %)	134 (93.1)	109 (92.4)	25 (96.2)	0.690
Perineural invasion (n, %)	118 (81.9)	96 (81.4)	22 (84.6)	0.472
Histologic grading system (n, %)				
Grade 1	15 (10.9)	11 (9.6)	4 (17.4)	0.195
Grade 2	71 (51.8)	57 (50)	14 (60.9)	
Grade 3	51 (37.2)	46 (40.4)	5 (21.7)	
Resection margin R1 (n, %)	10 (6.5)	6 (4.7)	4 (14.8)	0.073
T stage (n, %)				
T1	9 (6.5)	9 (7.9)	0	0.062
T2	63 (45.3)	50 (43.9)	13 (52)	
T3	64 (46)	54 (47.4)	10 (40)	
T4	3 (2.2)	1 (0.9)	2 (8)	
Nodal status (n, %)				
N0	29 (20.9)	23 (20.2)	6 (24)	0.504
N1	51 (36.7)	40 (35.1)	11 (44)	
N2	59 (42.4)	51 (44.7)	8 (32)	

**Fig. 1:**
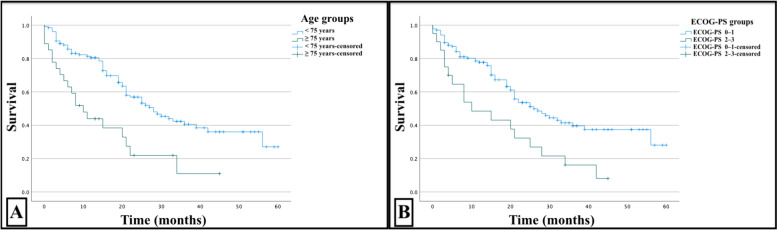
Survival curve (panel **A**) comparing < 75 years versus ≥ 75 years old patients and survival curve (panel **B**) comparing patients according to Eastern Cooperative Oncology Group Performance Status (ECOG-PS) scores

The overall median survival for the entire cohort was 25 months, with a range from 0 to 60 months. Group B had a cumulative median survival of 10 months (range 0–45), whereas Group A had a median survival of 28 months (range 0–60), with a statistically significant difference (p < 0.001), as shown in Fig. 1.

To obtain comparable numbers, patients were grouped into ECOG-PS 0–1 and ECOG-PS 2–3 groups according to their performance status. Figure 1 shows that the patients in the ECOG-PS 0–1 group had an overall median survival of 26 months (range 0–60), while those in the ECOG-PS 2–3 group had a median survival of 10 months (range 0–45), with a statistically significant difference (p = 0.003).

**Fig. 2:**
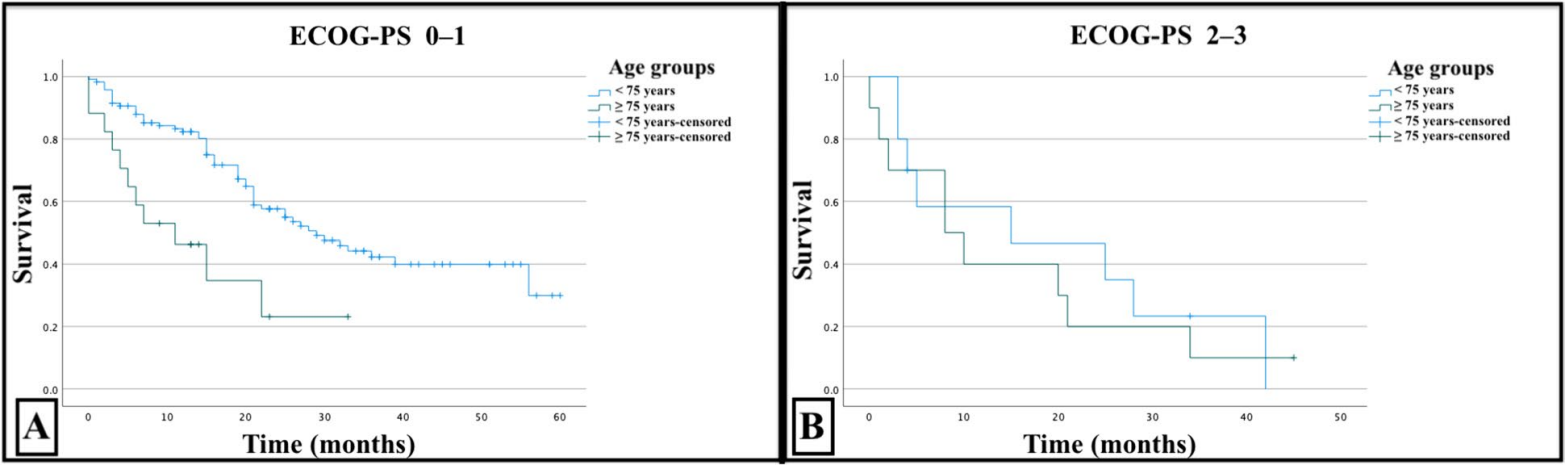
Survival curve comparing < 75 years versus ≥ 75 years old patients stratified according to ECOG-PS 0–1 (panel **A**) and ECOG-PS 2–3 (panel **B**)

When age groups were stratified according to the Eastern Cooperative Oncology Group (ECOG)-PS, the survival of patients aged ECOG-PS score of 0–1 < 75 years was 29 months, and the survival of patients aged ECOG-PS score of 0–1 ≥ 75 years was 11 months, which was a significant difference (< 0.001). However, the survival of patients with an ECOG-PS score of 2–3 and < 75 years was 15 months, and that of patients with an ECOG-PS score of 2–3 and ≥ 75 years was eight months; these differences were not statistically significant (p = 0.628). The ECOG-PS 0–1 stratified and ECOG-PS 2–3 stratified survival curves are shown in Fig. 2.

## Discussion

With the development of the healthcare system and the ease of access to medical treatment, life expectancy has increased significantly, and the proportion of older adults in the world has grown in parallel. In older patients, the incidence of pancreatic cancer with a poor prognosis is increasing, and the need for surgery is increasing. PD is one of the main treatment modalities for tumors and benign lesions in the pancreatic head and periampullary region. Even in high-volume surgery centers, the five-year survival rate after surgical resection of pancreatic cancer is not good. Therefore, offering this surgery to older patients is still a controversial issue, especially for the treatment of pancreatic cancer patients with a very poor prognosis. Studies have shown that age 75 years and older is an important factor affecting the prognosis of PD patients, but the results vary [30, 32, 33]. In this study, patients older than 75 years who underwent PD were compared with their younger counterparts, and no significant difference was found in terms of complications.

Conducting a thorough evaluation before surgery, providing appropriate care after surgery, managing pain effectively, ensuring proper nutrition, preventing delirium, and facilitating movement and rehabilitation are crucial for enhancing surgical outcomes in older patients and minimizing mortality rates and healthcare expenses. A multidisciplinary approach involving multiple specialties is mandatory for these optimizations. [9]

Performing surgical procedures on older individuals is particularly difficult because they have lower physiological reserves, impaired nutritional status, preexisting age-related health conditions, and cognitive impairment. As a result, these patients face a greater risk of postoperative complications and mortality than younger patients. A number of strategies have therefore been designed, including appropriate nutritional intervention. Currently recommended in clinical practice is the Enhanced Recovery After Surgery protocol and nutritional support [34, 35].

Emerging research indicates that frailty is a prevalent condition in the context of surgery and has substantial consequences after surgery. Frailty is an intricate, multifactorial, and recurring condition of decreased physiological capacity that leads to lower ability to recover and adapt in a person. It is marked by heightened susceptibility to various stressors. The concept of frailty is increasingly recognized as the most important condition in terms of health outcomes, particularly in individuals undergoing major surgery. Frail surgical patients have a greater risk of adverse outcomes, such as increased risk of postoperative complications and prolonged hospitalization. It can be challenging to determine the level of risk for surgery in this particular group, and relying solely on a person’s age may not provide enough information. In studies on this topic, the majority of the population undergoing abdominal surgery were found to be pre-frail or frail. Muscle strength measured by handgrip dynamometry can be used as a predictor of length of hospitalization in a surgical setting to determine frailty status [10, 12].

Aged patients have more comorbid diseases, and these conditions pose a risk for post pancreatectomy complications. Hypertension and cardiac comorbidities are risk factors for POPF and postoperative bleeding [18, 30, 33, 36, 37]. In an analysis of 6293 PD patients, approximately ten percent of whom were over 80 years of age, it was found that older patients also had higher rates of general complications and serious complications. Age, ASA classification, decreased functional status, a history of dyspnea, and a need for intraoperative transfusion were associated with worse postoperative outcomes [38]. In our study, older patients had worse performance scores and higher ASA scores. Hypertension was significantly more common in older patients, as expected, but there was no difference between the two groups regarding other comorbidities. However, patient selection bias is estimated in published studies [33]. Since our study was retrospective, we do not know the proportion of patients excluded due to severe comorbidities or age. Therefore, this may have affected our results.

According to a meta-analysis, the overall postoperative complication rate in patients aged 70 years and older who underwent PD was reported to be approximately 50%, and the rate of complications with a Clavien–Dindo grade ≥ 3 was approximately 30%, which is consistent with the results of our study. In this report, the mortality, postoperative complication, serious complication, intraoperative transfusion, and reoperation rates were significantly greater in patients aged 70 years and older than in younger patients [32]. In our study, the complication rates were similar between the two groups. The 30-day mortality rate was greater in older patients.

The indication for PD should not be based solely on age. In older patients, this decision should be made carefully, and meticulous perioperative management is key [2, 16, 17, 30, 35]. It is important to optimize comorbidities during the preoperative period and to prepare for possible postoperative deterioration. Older patients should be carefully selected for PD with appropriate clinical frailty classification. The physical and functional status of older patients should always be assessed and taken into account preoperatively, as hospitalization itself in this patient group may pose a risk due to functional decline and may result in the patient not being discharged home, leading to a reduced quality of life [6, 16, 38–40]. In our study, although the duration of postoperative hospitalization was similar, older patients required hospitalization for preoperative optimization, and the total length of hospitalization was longer.

In some studies comparing older patients with younger patients, no difference was found between the two groups when median survival was evaluated [33, 41]. However, in our study, the overall survival of older patients was worse. A patient’s ECOG performance status is an important prognostic factor for pancreatic cancer and is taken into account when making treatment decisions [42, 43]. In this study, patients with better ECOG-PS scores had longer survival than those with worse ECOG-PS scores. When age groups were stratified according to ECOG-PS, we found no significant difference in survival between the two groups in patients with worse performance scores (ECOG-PS 2–3). This emphasizes the importance of the patient’s performance status in terms of treatment selection.

Studies reporting the results of minimally invasive PD in older patients have shown that the benefits of this minimally invasive technique, such as shorter hospitalization and faster recovery, can also be utilized in older patients. Compared to studies on open methods, some studies have reported no difference in complications or mortality, while others have shown lower mortality outcomes. In general, the operation time is reportedly longer. Analyses revealed that laparoscopic PD is safe and feasible for older individuals and can be offered as an option for patients [28, 41, 44–46].

Given the frailty of the vast majority of older cancer patients and their increasing numbers, routine assessment of frailty in older cancer patients will be required to guide treatment and surgical decisions in these patients. Future research should focus on a comprehensive geriatric assessment using physical and functional parameters and their relationships with postoperative outcomes [9, 10, 40].

This study has several limitations. Our study was a single-center retrospective study with a small sample size. Selection bias may have been applied when recommending surgery to older patients, and this may have affected the results. A comparison of cancer-specific survival was more appropriate for this study, but the number of patients was too small when patients with missing data were excluded, so we could not provide this information. This information could have led to better conclusions. Although the ECOG-PS is widely used in oncology, it is a unidimensional functional score that is mostly physician-assessed, subjective and therefore prone to bias; therefore, it may fail to account for multimorbidity, frailty or cognition, but alternative scales that allow better patient selection could not be used due to the retrospective nature of the study [22].

## Conclusions

With the increasing aging population, patient selection for pancreaticoduodenectomy should not be based solely on age. This study demonstrated that pancreaticoduodenectomy is safe for patients over 75 years of age. In older patients, performance status and the optimization of comorbidities should be considered when deciding on a candidate’s suitability for surgery.

## Data Availability

The datasets generated during and/or analyzed during the current study are available from the corresponding author on reasonable request.

## References

[1] Ilic I, Ilic M (2022). International patterns in incidence and mortality trends of pancreatic cancer in the last three decades: a joinpoint regression analysis. World J Gastroenterol.

[2] Tan E, Song J, Lam S (2019). Postoperative outcomes in elderly patients undergoing pancreatic resection for pancreatic adenocarcinoma: a systematic review and meta-analysis. Int J Surg.

[3] Futagawa Y, Kanehira M, Furukawa K (2017). Study on the validity of pancreaticoduodenectomy in the elderly. Anticancer Res.

[4] Frakes JM, Strom T, Springett GM (2015). Resected pancreatic cancer outcomes in the elderly. J Geriatr Oncol.

[5] Uprak TK, Ergenc M (2023). Older is worse? Elderly patients who underwent gastrectomy: a single-center study. Ann Ital Chir.

[6] Scholer AJ, Marcus R, Garland-Kledzik M (2023). Validating biologic age in selecting elderly patients with pancreatic cancer for surgical resection. J Surg Oncol.

[7] Yazici H, Esmer AC, Eren Kayaci A (2023). Gastrıc cancer surgery in elderly patients: promising results from a mid-western population. BMC Geriatr.

[8] Ertekin SC, Cetindag O (2023). Assessment of surgical and quality-of-life outcomes between laparoscopic versus open inguinal hernia repair in geriatric patients. J Laparoendosc Adv Surg Tech A.

[9] Boccardi V, Marano L (2020). The geriatric surgery: the importance of frailty identification beyond chronological age. Geriatrics (Basel)..

[10] Marano L, Carbone L, Poto GE (2022). Handgrip strength predicts length of hospital stay in an abdominal surgical setting: the role of frailty beyond age. Aging Clin Exp Res.

[11] Fusario D, Neri A, Carbone L (2023). The Emergency Surgery Frailty Index (EmSFI) in elderly patients with acute appendicitis: an external validation of prognostic score. World J Surg.

[12] Han B, Li Q, Chen X (2019). Effects of the frailty phenotype on post-operative complications in older surgical patients: a systematic review and meta-analysis. BMC Geriatr.

[13] Ellis G, Gardner M, Tsiachristas A (2017). Comprehensive geriatric assessment for older adults admitted to hospital. Cochrane Database Syst Rev.

[14] Ergenc M, Uprak TK (2022). Esophagogastroduodenoscopy in patients aged 75 years and older: a single-center study. Cureus.

[15] Ergenç M, Uprak TK (2024). Lower gastrointestinal endoscopy in elderly: a single-center experience. South Clin Ist Euras.

[16] Casadei R, Taffurelli G, Silvestri S (2015). Is age a barrier to pancreaticoduodenectomy? An Italian dual-institution study. Updates Surg.

[17] Oliveira-Cunha M, Malde DJ, Aldouri A (2013). Results of pancreatic surgery in the elderly: is age a barrier?. HPB (Oxford).

[18] Sperti C, Moletta L, Pozza G (2017). Pancreatic resection in very elderly patients: a critical analysis of existing evidence. World J Gastrointest Oncol.

[19] El Nakeeb A, Atef E, El Hanafy E (2016). Outcomes of pancreaticoduodenectomy in elderly patients. Hepatobiliary Pancreat Dis Int.

[20] Yuan F, Essaji Y, Belley-Cote EP (2018). Postoperative complications in elderly patients following pancreaticoduodenectomy lead to increased postoperative mortality and costs. A retrospective cohort study. Int J Surg.

[21] Esmer AC, Tikici D, Tazeoğlu D (2022). Is the Whipple procedure safe and feasible in elderly patients?. Turkish Journal of Geriatrics.

[22] Simcock R, Wright J (2020). Beyond performance status. Clin Oncol (R Coll Radiol).

[23] Shin DW, Kim J (2020). The American Joint Committee on Cancer 8th edition staging system for the pancreatic ductal adenocarcinoma: is it better than the 7th edition?. Hepatobiliary Surg Nutr..

[24] Clavien PA, Barkun J, de Oliveira ML (2009). The Clavien-Dindo classification of surgical complications: five-year experience. Ann Surg.

[25] Bassi C, Marchegiani G, Dervenis C (2017). The 2016 update of the International Study Group (ISGPS) definition and grading of postoperative pancreatic fistula: 11 years after. Surgery.

[26] Wente MN, Bassi C, Dervenis C (2007). Delayed gastric emptying (DGE) after pancreatic surgery: a suggested definition by the International Study Group of Pancreatic Surgery (ISGPS). Surgery.

[27] Wente MN, Veit JA, Bassi C (2007). Postpancreatectomy hemorrhage (PPH)–an international study group of pancreatic surgery (ISGPS) definition. Surgery.

[28] Mederos MA, Starr S, Park JY (2023). Robotic versus open pancreaticoduodenectomy in elderly patients: a propensity score-matched analysis. HPB (Oxford).

[29] Tessman D, Chou J, Shebrain S (2022). Surgical outcomes of distal pancreatectomy in elderly patients. Am Surg.

[30] Fujinaga A, Kawasaki T, Hirashita T (2023). Technical and oncological safety of pancreatectomy for pancreatic cancer in older adults aged over 75 years versus younger adults: a single-center retrospective cohort study. Geriatr Gerontol Int.

[31] Dyussenbayev A (2017). Age periods of human life. Adv Soc Sci.

[32] Zhang W, Huang Z, Zhang J (2021). Safety and effectiveness of open pancreaticoduodenectomy in adults aged 70 or older: a meta-analysis. J Geriatr Oncol.

[33] Kim SY, Weinberg L, Christophi C (2017). The outcomes of pancreaticoduodenectomy in patients aged 80 or older: a systematic review and meta-analysis. HPB (Oxford).

[34] Marano L, Gu D, Dupre ME (2021). Nutrition and aging: surgical issues. Encyclopedia of Gerontology and Population Aging.

[35] Ergenc M, Karpuz S, Ergenc M (2021). Enhanced recovery after pancreatic surgery: a prospective randomized controlled clinical trial. J Surg Oncol.

[36] Uggeri F, Nespoli L, Sandini M (2019). Analysis of risk factors for hemorrhage and related outcome after pancreatoduodenectomy in an intermediate-volume center. Updates Surg.

[37] Casadei R, Ricci C, Lazzarini E (2014). Pancreatic resection in patients 80 years or older: a meta-analysis and systematic review. Pancreas.

[38] de la Fuente SG, Bennett KM, Pappas TN (2011). Pre- and intraoperative variables affecting early outcomes in elderly patients undergoing pancreaticoduodenectomy. HPB (Oxford).

[39] Paiella S, De Pastena M, Esposito A (2022). Modified frailty index to assess risk in elderly patients undergoing distal pancreatectomy: a retrospective single-center study. World J Surg.

[40] Handforth C, Clegg A, Young C (2015). The prevalence and outcomes of frailty in older cancer patients: a systematic review. Ann Oncol.

[41] Kim JS, Choi M, Kim SH (2022). Safety and feasibility of laparoscopic pancreaticoduodenectomy in octogenarians. Asian J Surg.

[42] Tas F, Sen F, Odabas H (2013). Performance status of patients is the major prognostic factor at all stages of pancreatic cancer. Int J Clin Oncol.

[43] Isaji S, Mizuno S, Windsor JA (2018). International consensus on definition and criteria of borderline resectable pancreatic ductal adenocarcinoma 2017. Pancreatology.

[44] Zhu J, Wang G, Du P (2021). Minimally invasive pancreaticoduodenectomy in elderly patients: systematic review and meta-analysis. World J Surg.

[45] Wang Q, Chen C, Li H (2022). Laparoscopic pancreaticoduodenectomy in elderly patients: systematic review and meta-analysis. Front Surg.

[46] Bartos A, Mărgărit S, Bocse H (2022). Laparoscopic pancreatoduodenectomy in elderly patients: a systematic review and meta-analysis. Life (Basel).

